# Two novel mutations in *ADAMTS13* in a Chinese boy with congenital thrombocytopenic purpura: a case report

**DOI:** 10.1186/s12881-020-00996-1

**Published:** 2020-03-20

**Authors:** Ling Hou, Yue Du

**Affiliations:** Pediatric Nephrology Department, Shengjing Hospital of China Medical University, No.36 Sanhao Street Heping District, Shenyang, 110004 Liaoning China

**Keywords:** Congenital thrombotic thrombocytopenic purpura (cTTP), *ADAMTS13*, Gene mutation

## Abstract

**Background:**

Mutations in the *ADAMTS13* gene family have been reported to cause congenital thrombotic thrombocytopenic purpura (cTTP), a rare disease characterized by thrombocytopenia and hemolytic anemia. Nearly 150 causative mutations in *ADAMTS13* have been identified; however, only a few of them were detected in Chinese patients.

**Case presentation:**

A 5-year-old Chinese boy presented with history of thrombocytopenic purpura, hemolytic anemia, and renal injury since the neonatal period. Gene analysis revealed two novel mutations in *ADAMTS13*: a missense mutation 332G > A (p:Gly111Glu) in exon4 and a nonsense mutation 3121C > T (p:Gln1041stop) in exon 24. Genetic analysis of his parents confirmed the heterozygous nature of the mutations.

**Conclusion:**

We report two novel mutations in *ADAMTS13* (332G > A, 3121C > T) in a Chinese boy. These two mutations may lead to early onset of cTTP and severe symptoms.

## Background

Thrombotic thrombocytopenia purpura(TTP) is a rare, life-threatening disease. The condition is caused by the formation of unusually large von Willebrand factor (vWF) multimers (ULVWFMs) owing to the deficiency of vWF cleaving protease (VWF-CP) [1]; this causes abnormal platelet aggregation and results in thrombocytopenic purpura, hemolytic anemia, and microangiopathy in vital organs such as the brain and kidney [1, 2]. Most cases of TTP have acquired TTP (aTTP) caused by the presence of antibodies or inhibitors of VWF-CP, while cases of congenital TTP (cTTP) caused by gene defect are relatively rare [3]. Levy et al. (2001) reported that the gene that encodes for VWF-CP belongs to the *ADAMTS13* family [4]. Since then, more than 80 gene mutations in *ADAMTS13* causing cTTP have been reported [5]; most of these were missense mutations, while others were nonsense, insertion, deletion, frameshift, or splicing mutations [6]. However, to the best of our knowledge, only a few Chinese cases of cTTP have been reported with mutations in *ADAMTS13* [7–10]. Here we report a Chinese boy with two novel mutations in *ADAMTS13*: one missense mutation in exon 4 and a nonsense mutation in exon 24. The clinical course was marked by recurrent thrombocytopaenic purpra, haemolytic anaemia, and renal injury since the neonatal period.

## Case presentation

A 5-year-old male Chinese neonate developed jaundice and dark urine at 18 h after birth. Laboratory investigations revealed hemolytic anemia, thrombocytopenia, and impaired renal function. Hematological examination revealed hemoglobin (Hb) 105 g/L (normal reference range, 180–190 g/L), platelet count 34 × 10^9^/L (normal reference range, 34 × 10^9^/L), fragmented red blood cells (helmet- or drop-like morphology) in blood smear examination, and negative Coomb’s test. The biochemistry parameters were: total bilirubin (TBILI) 479.5 μmol/L (normal reference range, 3.4–20.5 μmol/L); unconjugated bilirubin (UCB) 458.8 μmol/L (normal reference range, 3.4–11.9 μmol/L); lactate dehydrogenase (LDH) 1181 U/L (normal reference range, 80–285 U/L); serum creatinine (Scr) 156.5 μmol/L (normal reference range, 59–104 μmol/L), blood urea nitrogen (BUN) 13.89 mmol/L (normal reference range, 3–9.2 mmol/L). He was diagnosed with hemolytic uremic syndrome (HUS) and achieved remission upon treatment with exchange blood transfusion. At the age of 4 years and 5 months, the boy experienced a relapse with pallor, petechiae, and dizziness; laboratory investigations showed severe thrombocytopenia (platelet count: 8 × 10^9^/L), hemolytic anemia (Hb 72 g/L; schistocytosis on blood smear examination), and impaired renal function (Scr 203.9 μmol/L; BUN 13.38 mmol/L). His symptoms relieved with plasma exchange; in addition, hypotensive drugs were administered for high blood pressure (as high as 180/100 mmHg). Five months later, he experienced second relapse with scattered haemorrhagic dots and platelet count of 16 × 10^9^/L; his symptoms were relieved with plasma exchange. The most recent relapse happened 4 months later with the symptoms of skin petechiae and dizziness. His platelet count was 18 × 10^9^/L, glomerular filtration rate (GFR) was 59.14 mL/min/1.73 m^2^ (normal reference range, 80–120 mL/min/1.73m^2^) and blood pressure was 175/107 mmHg. Moreover, he developed upper respiratory infection prior to the last two relapses. When the boy was referred to us at the last relapse, ADAMTS13 protein activity, inhibitors were examined by residual-collagen binding assay and *ADAMTS 13* gene analysis was performed. ADAMTS13 protein activity was 5.7% (normal range, 40–130%), while ADAMTS13 inhibitors were negative; in addition, two novel mutations in this gene were found, which confirmed the diagnosis of cTTP. Subsequently, the boy received prophylactic fresh frozen plasma (FFP) infusion every 2 weeks. He did not develop any relapse in the subsequent 2 months and showed improvement in renal function.

**Fig. 1:**
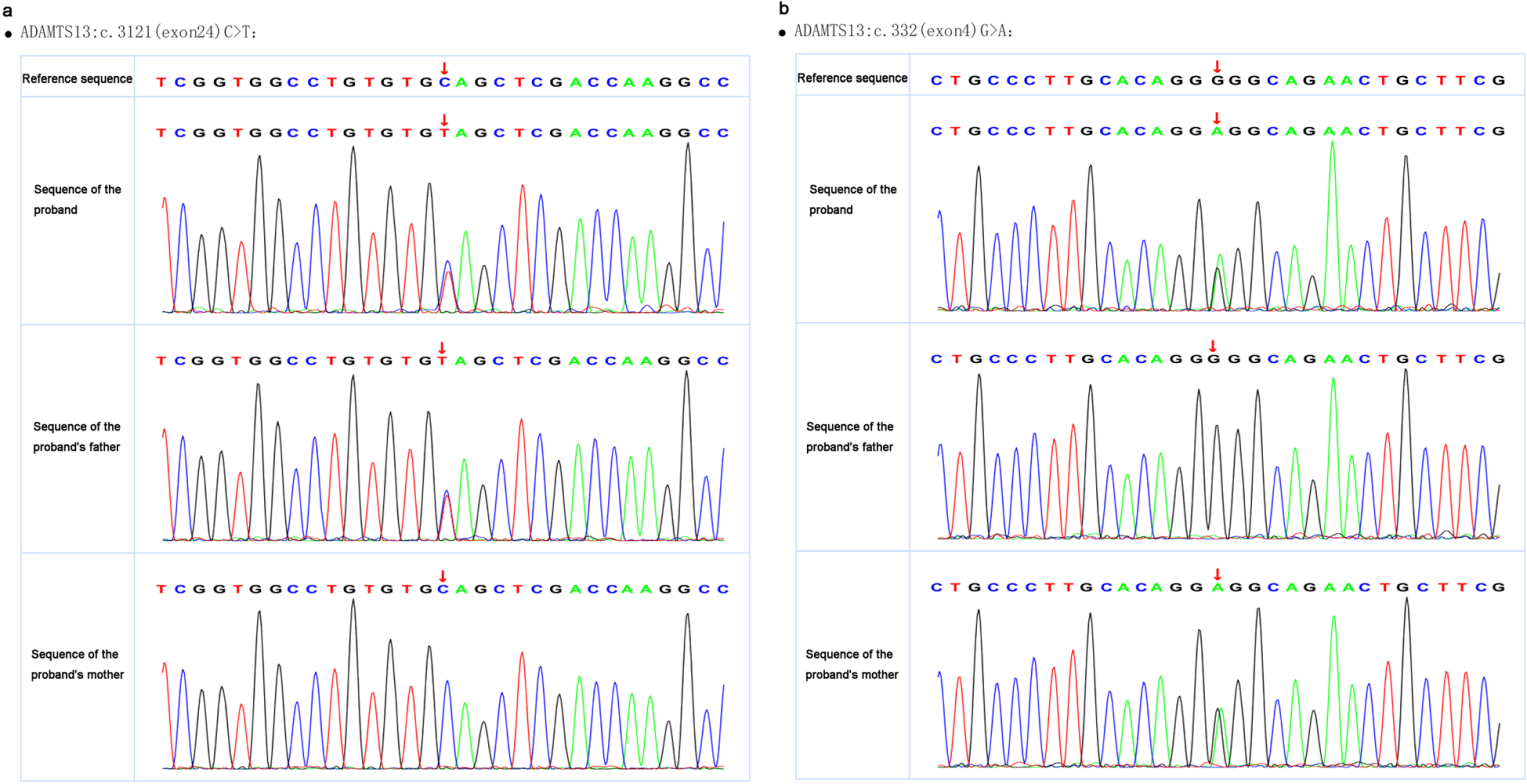
**a** 332C > A in exon4. **b** 3121C > T in exon24

Through polymerase chain reaction (PCR) amplification and direct sequencing of the 29 exons and intron boundaries of the *ADAMTS13* gene, two mutations (332G > A in exon4 and 3121C > T in exon 24) were found. The first one (332G > A) was a missense mutation involving exchange of glycine for glutamic acid (Gly111Glu), while the other (3121C > T) was a nonsense mutation involving exchange of glutamine for a termination codon (Gln1041stop) and a truncated protein that would form in this region. *ADAMTS13* gene of the boy’s parents were also analyzed, and the results indicated that the boy had inherited 332G > A mutation from his mother and 3121C > T mutation from his father. This proved that the nature of the mutations that caused cTTP was compound heterozygote mutation (Fig. 1). The parents were carriers of one each of these two novel mutations, and manifested no symptoms of this disease. Nevertheless, the vWF activity had not been detected due to the constraints at our department.

## Discussion and conclusions

Our patient with TTP had been misdiagnosed for a relatively long time. According to Assink et al. (2003) [2], TTP is usually characterized by the pentad of thrombocytopenia, hemolytic anemia, neurologic signs, renal injury, and fever. However, many patients may manifest oligosymptomatic forms, as observed in our patient; our patient had no fever or neurological signs, which made the diagnosis challenging. In 2014, Bendapudi et al. proposed the PLASMIC score to assess the risk of low ADAMTS13 protein activity and suspected TTP; the score is based on symptoms (hemolysis), history (cancer and organ transplant), and laboratory examination (Plt, MCV, Scr, and INR) [11]. According to this score, our patient could be considered as intermediate risk, which might demonstrate its advantages. Moreover, detection of ADAMTS13 inhibitor and *ADAMTS13* sequences may help confirm the diagnosis of TTP.

HUS and TTP have similar characteristics such as thrombocytopenia, nonimmune haemolytic anaemia, and multiorgan dysfunction; however, the two conditions are believed to be different disease entities [12]. Our patient showed signs of mild-moderate renal injury; this is in contrast to the patient reported by Schneppenheim [13], who developed acute renal failure. Other differentiating points from HUS include the lack of increase in fibrinogen levels and the absence of gastrointestinal symptoms throughout the disease course [1]. In addition, the level of ADAMTS13 protein activity and inhibitors are obviously lower in cTTP, as seen in our patient; this is different from HUS.

**Fig. 2:**
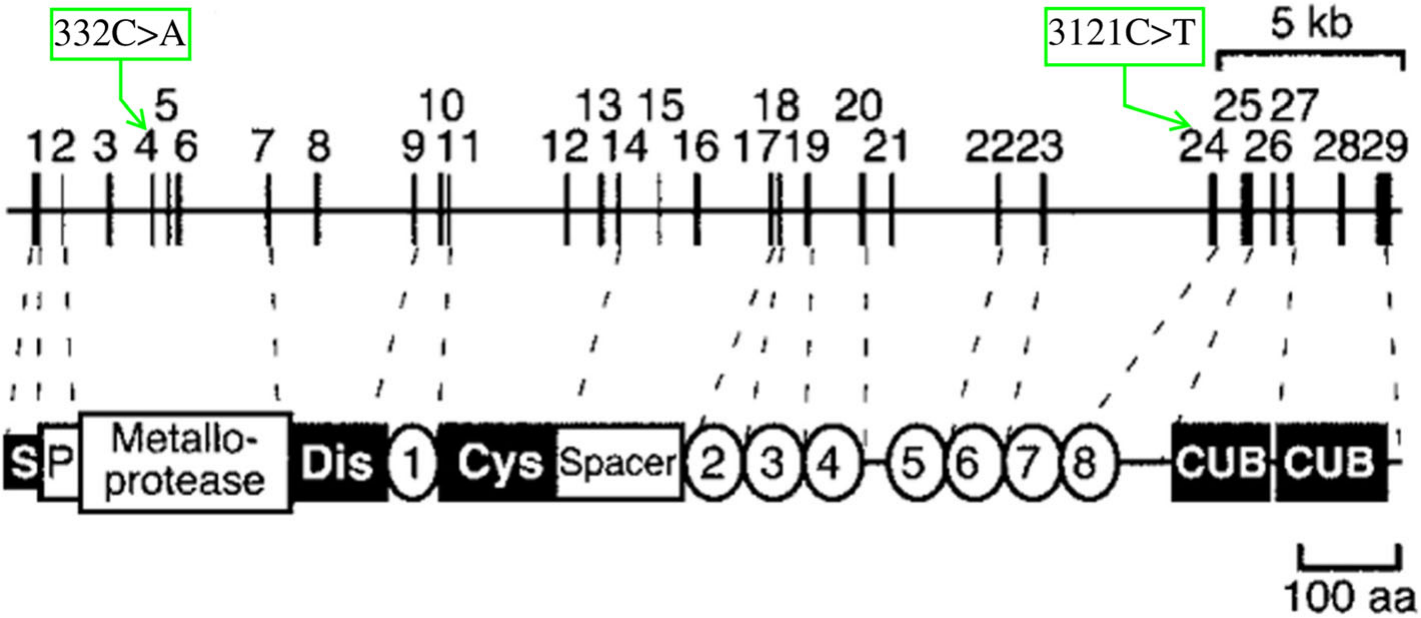
Schematic illustration of the structure of ADAMTS13.Exons 1–29 are shown to scale above the illustration of the protein structure. Dashed lines indicate the correspondence between the boundaries of exons and the boundaries of structural domains. S, signal peptide; P, propeptide; Dis, disintegrin-like; Cys, Cys-rich. TSP1 repeats are numbered 1–8

*ADAMTS13* is approximately 37 kb long and is located at chromosome 9q34. From its N terminus the encoded metalloprotease *ADAMTS13* comprises a signal peptide domain, a propeptide domain, a metalloprotease domain, a disintegrin like domain, a thrombombospondin type 1 repeat (TSP1) domain, a cysteine-rich domain, a spacer domain, seven additional TSP1 repeats, and two terminal complement C1r/C1s, Uegf, Bmp1 (CUB) domains [13]. Till date, mutations in *ADAMTS13* that cause cTTP have been found to affect nearly all these domains [14]. The missense mutation of 332G > A in exon4 is in the metalloprotease region, which correlates with the protease activity of ADAMTS13. More than 19 mutations have been found in this region; according to vivo and vitro studies, these may affect the ADAMTS13 function by reducing the secretion of or the cleavage activity of protease [15, 16]. According to a previous case report, the proximity of exon4 to calcium binding sites in aa82 and aa173 may explain the consequences of the mutation; the Gly111Glu caused by 332G > A in our patient is also located in close proximity to these two sites in this region [17]. The other mutation was a nonsense mutation in the TSP1–7 region; the effects of mutations in this region have not been clearly reported (Fig. 2). Kazuyoshi et al. [18] found a species of naturally variant mouse with *ADAMTS13* lacking TSP1–7, 8 and two CUB domains;in vitro, these truncated recombinant ADAMTS13 also showed VWF-CP activity. Nevertheless, the reported cases with mutations, especially nonsense mutations in TSP1–7, 8 domains in *ADAMTS13*, often manifested severe clinical symptoms [1, 4, 19–21]. Besides, in their in vivo and vitro studies, both Donadelli and Camilleri found that mutations in the TSP1–7 domains of *ADAMTS13* may affect the catalytic activity or secretion of VWF-CP [22, 23].

Our patient showed early onset of symptoms such as jaundice, thrombocytopenia, and anaemia. However, there are several case reports where in the patient suffered the first episode during adolescence, adulthood, or even in old age; moreover, the disease severity also showed considerable variability [24–26]. The heterogeneity in phenotype is not clearly explained by the difference in genotype. A patient with a homozygous mutation in exon4 was reported by Meyer et al. [17];they suggested that mutations in the metalloprotease domain may lead to a first TTP bout in young adulthood, rather than in early childhood. Therefore, in comparison to previous reports, the neonatal onset in our case may be attributable to the nonsense mutation in TSP1–7 [24–27];however, the phenotype-genotype correlation of *ADAMTS13* has not been established partly because of the rarity of homozygous cases [5, 20, 28–32].

We reported a Chinese boy with cTTP who presented with recurrent thrombocytopenia, haemolytic anaemia, and renal injury. Two novel mutations, a missense mutation 332G > A in exon4 and a nonsense mutation 3121C > T in exon 24, were detected in this patient. The nonsense mutation of 3121C > T may have contributed to the early disease onset in the neonatal period.

## Data Availability

The gene sequencing data of *ADAMST13* is stored in NCBI Sequence Read Archive (SRA) (accession number: SRR11248993).

## Abbreviations

cTTP: Congenital thrombotic thrombocytopenic purpura
TTP: Thrombotic thrombocytopenia purpura
vWF: von Willebrand factor
ULVWFMs: Unusually large von Willebrand factor (vWF) multimers
VWF-CP: vWF cleaving protease
aTTP: acquired TTP
cTTP: congenital TTP
Hb: Hemoglobin
TBILI: Total bilirubin
UCB: Unconjugated bilirubin
LDH: Lactate dehydrogenase
Scr: Serum creatinine
BUN: Blood urea nitrogen
HUS: Hemolytic uremic syndrome
GFR: Glomerular filtration rate
FFP: Fresh frozen plasma
PCR: Polymerase chain reaction
TSP1: Thrombombospondin type 1 repeat
CUB: C1r/C1s, Uegf, Bmp1
